# Wild Birds Pose Unique Food Safety Threats in the US Southeast

**DOI:** 10.3390/ani15192813

**Published:** 2025-09-26

**Authors:** Sofia Varriano, Jared C. Smith, Olivia M. Smith, Pedro A. P. Rodrigues, Zachary Snipes, Kerrie Roach, Joshua L. Dawson, Justin Shealy, Laurel L. Dunn, Nikki W. Shariat, William E. Snyder

**Affiliations:** 1 Department of Entomology, University of Georgia, Athens, GA 30602, USA; smitho17@msu.edu (O.M.S.); pedro.rodrigues@uga.edu (P.A.P.R.); wesnyder@uga.edu (W.E.S.); 2 Departments of Population Health and Microbiology, University of Georgia, Athens, GA 30602, USA; jared.smith1@uga.edu (J.C.S.); nikki.shariat@uga.edu (N.W.S.); 3 Department of Horticulture, Michigan State University, East Lansing, MI 48823, USA; 4 Ecology, Evolution, and Behavior Program, Michigan State University, East Lansing, MI 48823, USA; 5 Department of Plant Industry, Clemson University Extension, Charleston, SC 29401, USA; zbsnipe@clemson.edu (Z.S.); kwalker@g.clemson.edu (K.R.); 6 College of Agriculture, Family Sciences, and Technology, Fort Valley State University Extension, Fort Valley, GA 31030, USA; dawsonj01@fvsu.edu; 7 College of Agricultural and Environmental Sciences, University of Georgia Extension, Athens, GA 30602, USA; justin1@uga.edu; 8 Department of Food Science and Technology, University of Georgia, Athens, GA 30602, USA; laurel.dunn@uga.edu; 9 Center for Food Safety, University of Georgia, Griffin, GA 30223, USA

**Keywords:** agroecosystems, foodborne pathogens, birds, ecosystem disservices, landscape ecology, *Salmonella*, food safety

## Abstract

Natural areas near farmland can provide refuge for birds that contribute to natural pest control. However, birds can endanger food safety by defecating on or near produce. Work in the western US suggests that *Campylobacter* spp. are the potential foodborne pathogens most commonly associated with wild birds and that pathogen prevalence is higher in landscapes dominated by animal agriculture. However, relatively little is known about other fresh-market-produce growing regions. Working on produce farms in the Southeastern US, we characterized bird communities, tested bird feces deposited on crop foliage for *Campylobacter* and *Salmonella*, and searched for landscape features associated with heightened bird-associated food safety risks. We found that bird communities on farms were generally similar across ecoregions. *Campylobacter* was never detected from bird feces deposited on crop foliage, but *Salmonella* was detected in 8.6% of fecal samples. *Salmonella* prevalence in crop-surface-collected bird feces was highest when farms also produced livestock and when wetland cover was prevalent in the landscape. Overall, our results suggest that on-farm livestock production may be an indicator of bird-associated food safety risks in the Southeast, as in the West. We suggest there may be some similarities, but important differences, in food safety risks posed by birds in different US produce growing regions.

## Simple Summary

Wild birds may endanger food safety when they defecate onto fresh produce. These risks have been primarily studied in the western US, where *Campylobacter* is the most common bacterial foodborne pathogen associated with birds, and pathogen prevalence increases in bird feces in crop fields near cattle production. To understand the food safety risks posed by birds in the US Southeast, we collected bird feces from produce fields and tested them for the presence of *Salmonella* and *Campylobacter*. *Campylobacter* was never detected in bird feces on crop plants, while *Salmonella* detection (in 8.6% of fecal samples) was most common on farms within landscapes with abundant wetlands that also had livestock. Birds may pose different food safety risks in the Southeast than elsewhere.

## 1. Introduction

A growing human population presents ever-greater challenges in conserving biodiversity while also maintaining robust food production [1]. Incorporation of natural areas into farmlands can help mitigate this conflict, as uncultivated areas can provide refuge for pollinators, predators, and decomposers that benefit crops [2,3,4]. For example, birds in farming landscapes can move from natural habitats into fields to consume herbivorous pests, strengthening natural pest control [5]. For many years, food safety was thought to run counter to these broader trends, with natural areas attracting birds and other wildlife that might defecate on nearby crops, risking contamination with *Salmonella*, *Campylobacter*, or other bacteria that cause foodborne illness in humans [6]. These concerns led to pressure on fresh-market-produce growers to remove natural habitats from farms to deter wildlife and lessen food safety risks, despite the likely costs to other beneficial ecosystem services [7].

More recently, extensive work, largely conducted on produce farms in the western US, has provided clear evidence that natural habitats can act to reduce, rather than enhance, food safety risks [8,9]. For example, Smith et al. (2020) [10] found that foodborne pathogen prevalence in produce was reduced on farms embedded in more natural landscapes. In contrast, farming in landscapes with more dense cattle (*Bos taurus*) production and more actively grazed lands are correlated with increased food safety risks [11]. This may be because farming landscapes with greater natural habitat support bird species that are less likely to interact with domesticated livestock and therefore reduce the opportunities to encounter bacterial foodborne pathogens [11,12]. However, it remains unclear whether the drivers of bird-associated food safety risks seen along the US west coast are the same as those found in other produce-growing regions [13,14]. Thus, we are unsure whether we can draw general recommendations for all growers from the lessons learned in the west, or if, instead, it will be necessary to develop region-specific recommendations for managing on-farm food safety risks posed by birds [15].

The southeastern US houses a growing fresh-market produce industry, with fields embedded in ecoregions that are distinctly different from the western part of the country and often interspersed among wetlands and other natural habitats [16]. Surveys of potential foodborne pathogens associated with birds in this region have included wading birds, songbirds, and others, usually in non-agricultural habitats [17,18,19]. Bird communities found in or near produce fields, and their associated potential foodborne pathogens, have been relatively unexplored in the Southeast [13]. Here, we seek to fill this knowledge gap through two complementary objectives. First, working on the farms of cooperating produce growers, we conducted surveys of bird species associated with crop fields, to describe which species were present and whether bird assemblages differed among ecoregions, landscapes, and farm production types. Second, we searched fields for bird feces on crop plants, which were collected to determine *Salmonella* and *Campylobacter* prevalence. We then modeled landscape and local attributes (e.g., percent of wetlands and other natural cover types, presence of cattle or other livestock on the farm) that might be associated with a higher risk of bird feces being contaminated with foodborne bacteria. Our ultimate goal was to allow a comparison between the ecology of bird-associated foodborne pathogen transmission in the Southeast with what is known from the far-better-studied western US, with the goal of developing widely applicable plans to mitigate food safety risks posed by birds.

## 2. Materials and Methods

Data collection included (1) point count surveys of birds in or adjacent to produce fields and (2) local- and landscape-scale data that were used to examine correlates of *Salmonella* prevalence (*Campylobacter* was never detected, see below, and thus was not modeled) in crop-surface-collected bird feces. Genetic characterization of *Salmonella* isolates collected as part of the field work reported here was reported in a companion study, Smith et al. (2023) [20]. Here, we expand upon Smith et al. (2023) [20] by examining (1) how bird communities in or near produce fields vary with ecoregion, landscape, and farm management and (2) how *Salmonella* prevalence in wild bird feces correlates local- and landscape-attributes.

### 2.1. Study Sites

**Figure 1:**
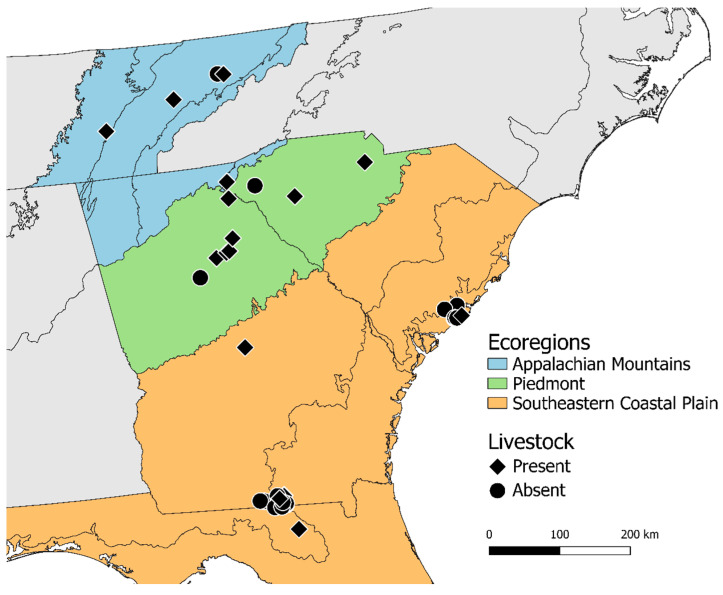
Map of farms included in our study (*n* = 43) from which we collected fecal samples. Color corresponds to ecoregion (blue = Appalachian Mountains, green = Piedmont, and orange = SE Coastal Plains), and shape corresponds to whether livestock were also produced on-farm (diamond = present, circle = absent). Farms included in this study are only those surveyed from May-August (*n* = 43), as opposed to those in Smith et al. (2023) [20] (*n* = 45).

Across 2 years, we surveyed bird communities and collected bird feces from crop foliage on 43 farms in north Florida (*n* = 3), Georgia (*n* = 26), South Carolina (*n* = 10), and eastern Tennessee (*n* = 4), USA. We visited each farm 1–5 times across the 2-year study, between May-August 2021 and 2022 (total visits = 85; mean/farm = 2) (Figure 1) [20]. These farms spanned a key produce-growing region of the southeastern US, an industry now worth ca. $19.5 billion per year that delivers fresh-market produce to much of the eastern US [16]. Regional fruit and vegetable production spans at least three distinct ecoregions—Appalachian Mountains, Piedmont, and Coastal Plains (Figure 1)—which might house distinct wild bird communities [21]. Farming landscapes in our study region are often diverse, with production fields interspersed with pine plantations, wetlands, and other less intensively managed habitats (Table S1). This mosaic landscape provides ample opportunity for birds to regularly forage in, or travel above, produce fields, and for farm-bird communities to be influenced by surrounding natural habitats (e.g., [22]). Farms ranged from large monoculture fields (*n* = 26) to smaller, highly diversified operations (*n* = 17) that produced many non-vegetable crops [e.g., ornamentals, apples (*Malus domestica*), and pecans (*Carya illinoinensis*)] alongside various vegetables [e.g., onions (*Allium cepa*), beans (*Phaseolus vulgaris*), and squash (*Cucurbita pepo*)]. The average farm size in this study was 22.1 ha ± 7.5 (SE; range 0.61–94.3 ha) (Table 1). Organic-only practices accounted for 32.6% (14/43) of farms, while the remaining farms used either conventional practices or a combination of both organic and conventional. Additionally, livestock was produced on 12 (27.9%) of these farms. Livestock produced on-farm included chickens (*Gallus gallus*; 8 farms), cattle (*Bos taurus*; 4), horses (*Equus caballus*; 3), goats (*Capra hircus*; 2), pigs (*Sus domesticus*; 1), donkeys (*Equus asinus*; 1), and/or ducks (*Anatidae* spp.; 1; Table 1).

### 2.2. Bird Fecal Sample Collection

We collected bird feces from crops that produce fruit or vegetables above ground (e.g., on stakes or trellises) to avoid collecting fecal samples from non-bird animals and limit contamination from splash-back or ground contact. Additionally, we selected crops that are commonly eaten uncooked, as these represent the largest food safety risk to consumers. Our selected crops included primarily tomatoes (*Solanum lycopersicum*), cucumbers (*Cucumis sativus*), bell peppers (*Capsicum annuum*), eggplants (*Solanum melongena*), and grapes, both table and wine (*Vitis* spp.). Fecal samples were collected from selected produce fields on each farm between sunrise and 11 a.m. as described in Smith et al. (2023) [20]. Briefly, the perimeter of each field was surveyed for bird feces deposited on the leaves of plants; when field size allowed (20 ha or less), the interior was also sampled by walking in a step-wise pattern among the rows. Fecal samples were scored as either “dry” or “moist” based on visual appearance as an approximate measure of whether the sample was relatively freshly deposited (estimated within 3 h since deposition) or older. We found that this moist/dry designation was a strong predictor of whether Salmonella could be detected in a fecal sample, indicating that drier samples had likely been sitting for enough time that DNA had degraded or the pathogens had become nonviable [20]. Feces were collected by clipping leaves around the entire feces into a resealable plastic bag filled with 2 mL buffered peptone water (BPW) recovery media. Plastic bags were placed on ice after collection until processing in the lab, which occurred within 24 h [20]. We finished collection at each farm after either inspecting every plant or searching for 5 h (until 11:00 a.m.), whichever occurred first. The number of fecal samples collected during a visit ranged from 0 to 30.

### 2.3. Characterizing Bird Communities

Bird communities on each farm were surveyed via standardized point-counts (e.g., [12]). One point-count was performed for every 10 ha of sampled field when field conditions and harvesting schedules allowed (total point count locations = 106, mean/farm = 2.7). Point-counts were conducted on still, clear mornings between 6 and 10 am, all by the same observer. Points were positioned near the edges of fields to overlap with fecal sampling areas while still capturing birds moving in and out of produce fields. Points on the same farm were at least 200-m apart. All birds seen and heard within a 100-m radius during a 10-min period were recorded, along with the habitats they were observed in. Birds flying overhead were excluded unless they were a species that forages aerially (e.g., Barn Swallows, *Hirundo rustica*), in which case a note was made that they were “aerial foraging”.

We used land cover data from the National Land Cover Database [23] to test for associations between land cover and bird communities. Land cover was categorized as “open water” (NLCD code 11), “barren” (NLCD code 31), “natural habitat” (NLCD codes 41-43, 51-52, 71-74, 90, 95), “developed” (NLCD codes 21-24), and “agricultural” (NLCD codes 81-82). We also tested “wetlands” (NLCD codes 90, 95) separately from other natural habitat due to the number of wetland-associated bird species that had *Salmonella* detected in their feces in prior work in the system [20].

We determined a biologically relevant landscape scale by weighting the home range of each bird species with known sizes, gathered from Birds of the World Online [24], by the relative abundance of recorded individuals in our point-count surveys (e.g., [10]). This resulted in a 4.5 km radius, which was subsequently used to generate land cover data from the center of each farm. Farm size was calculated by tracing around the edge of each farm in QGIS v3.28.0 and measuring the area of the subsequent polygon.

Additionally, for each farm, we calculated a series of values representing both natural habitat configurational and compositional heterogeneity in FRAGSTATS 4.2 (Table S1) [25] following [10]. Landscape heterogeneity variables were highly correlated (Figure S1), so we used only interspersion and juxtaposition index of natural cover types in our models. Livestock were recorded as “on-farm” (1) if they were present on-farm the day of sampling; “nearby” (2) if they were present within 250 m of the farm; or “absent” (0) if they were neither on-farm nor within 250 m of the farm the day of sampling.

A non-metric multidimensional scaling (NMDS) plot with a Bray–Curtis dissimilarity matrix was used to examine how bird communities varied across farms. Analyses were conducted in the R package vegan (v2.6.4) [26]. Species abundances were averaged across visits and point counts for each farm. We tested for the relationships between proportion of land cover values listed above and species abundances in the community (Bray–Curtis distances) with a series of Mantel tests, using livestock presence [“on-farm” (1) and “nearby” (2) as described above] and farm size as strata for permutations. Associations were examined using Spearman’s correlation coefficients. We also tested whether bird communities varied significantly with ecoregion, accounting for farm size and livestock presence, using a series of ANOSIM tests (α = 0.05, permutations = 999) from the R package vegan. Indicator species were identified for each ecoregion using the function multipatt from the R package indicspecies (α = 0.05) [27].

### 2.4. Linking Salmonella Prevalence to Local and Landscape Factors

We tested all foliage-collected bird fecal samples for the presence of *Salmonella* and *Campylobacter* by both culture and PCR screening. *Salmonella* testing procedures are described in detail by [20]. Briefly, samples and recovery media were homogenized prior to processing. For *Salmonella*, 750 µL of sample and media homogenate were added into 9.25 mL of BPW (Hardy Diagnostics, Springboro, OH, USA. Following pre-incubation at 42 °C for 24 h, 1 mL and 0.1 mL of culture were sub-cultured in parallel into 9 mL Tetrathionate (TT, Neogen, Diagnostics, MI, USA) and 9.9 mL Rapport-Vassiliadis (RV, Hardy Diagnostics, OH, USA) selective enrichment broths, respectively. Cultures were incubated for an additional 24 h at 37 °C before being streaked onto Xylose Lysine Tergitol-4 agar plates (XLT-4, Hardy Diagnostics, OH, USA). Plates were incubated at 37 °C for 24–48 h before being inspected for black colonies indicating the presence of *Salmonella*. For *Campylobacter*, 750 µL of the sample and media homogenate were added into 8.5 mL of Bolton’s broth (BB, Hardy Diagnostics, OH, USA) before placing samples in a sealed bag filled with blood-gas atmosphere and mixing gently. Samples were incubated at 42 °C for 48 h before being streaked onto Cefex agar plates (Hardy Diagnostics, OH, USA). Plates were placed in a bag filled with blood gas atmosphere at a concentration of 5% oxygen, 5% carbon dioxide, and balanced with nitrogen (Airgas, Radnor, PA, USA) and incubated at 42 °C for another 48 h. Plates were then visually inspected for *Campylobacter* colonies.

For PCR screening for both *Campylobacter* and *Salmonella*, total genomic DNA was extracted from 500 µL of the remaining unincubated sample homogenate using the Genome Wizard kit (Promega, Madison, WI, USA). Extracted DNA was aliquoted into multiple tubes to limit freeze–thaw degradation, and these were stored at −20 °C until PCRs were performed. DNA was tested for the presence of PCR inhibitors using an internal amplification control (IAC) PCR [28]. For *Salmonella* screening, we used an invA PCR [29] as described in [20]. For *Campylobacter* screening, we first ran a general 16S PCR [30]. Each 16S reaction contained 3 µL 10× reaction buffer, 100 mM dNTPs, 0.3 µM F primer, 0.3 µM R primer, 1 U Taq, and 2 µL DNA. Sterile water was added until the final reaction volume was 30 µL. Amplification occurred with the following cycle: denaturation at 95 °C for 2 min; 24 cycles of amplification (95 °C for 30 s, 51 °C for 30 s, 68 °C for 30 s); and a final extension at 68 °C for 2 min. PCR products were visualized on a 1.5% agarose gel. *C. jejuni*, *C. coli*, and *C. lari* strains were included as a positive control.

We focused on *Salmonella* prevalence in our models examining how local livestock and landscape factors impact wild bird-mediated food safety risks because we did not detect *Campylobacter* in any of the fecal samples. We considered a sample positive for *Salmonella* if either culture or PCR returned a positive result. *Salmonella* prevalence was significantly higher in moist [15.8% (45/285)] than dry samples [14.5%, (60/415)] (χ^2^ = 30.44, *p* < 0.01). Because our ability to detect *Salmonella* was higher when samples were moist [20], only moist samples (*n* = 285) were considered in statistical models.

We used a series of generalized linear mixed effects models (GLMMs) with a binomial distribution fit in the R package glmmTMB [31] to test the relationship between *Salmonella* prevalence and (1) proportion developed, natural, agricultural, open water, and wetland land cover within 4.5 km of farms; (2) natural habitat heterogeneity; and (3) cattle, chicken, or any (cattle, chicken, or other) livestock presence on farms. Because ecoregion was not significantly associated with differences between bird communities, we did not include ecoregion in these models. Further, we counted livestock in these models as either “on-farm” (1) or “absent from farms” (0,2).

We considered both additive and interactive fixed effects between individual landscape variables and local livestock variables that we hypothesized would impact *Salmonella* prevalence (Table S2). Farm visit was treated as a random effect nested within farm for all models, and year was included as a fixed effect in all models. Continuous variables were z-score transformed prior to analysis. Multicollinearity was assessed with the performance package in R [32]; any models with covariates that had a variance inflation factor (VIF) ˃ 5 were not considered. We considered models well-supported based on the criterion of ΔAICc ≤ 2 from the most well-supported model [33]. Model predictions were generated for each model, weighted by the relativized AICc model weight, and then averaged to generate an overall *Salmonella* prevalence prediction. Weighted variance was calculated using the *wtd.var* function from the R package Hmisc [34]. All statistical analysis was performed in R (v. 4.3.2, 4.4.2) [35].

## 3. Results

### 3.1. Bird Community Analysis

We identified 859 birds from 47 different species during point counts (Table S3). The most abundant species detected included the Mourning Dove (*Zenaida macroura*), Rock Pigeon (*Columba livia*), Barn Swallow, and House Finch (*Haemorhous mexicanus*). Five of the detected species—Eurasian Collared-Dove (*Streptopelia decaocto*), European Starling (*Sturnus vulgaris*), House Finch, House Sparrow (*Passer domesticus*), and Rock Pigeon—were not native to the study area.

**Figure 2:**
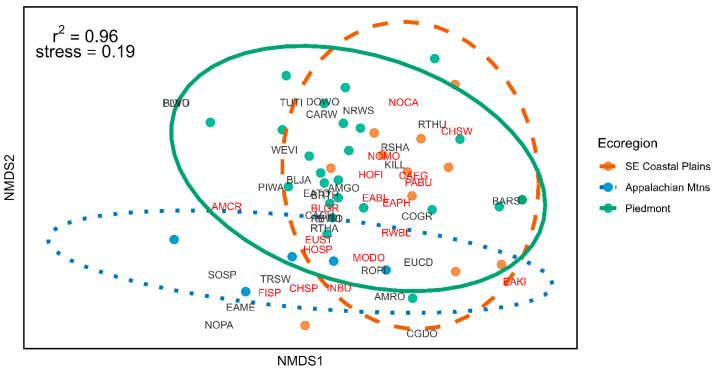
Bird species, given by four-letter alpha codes (text), were not noticeably clustered by ecoregion. Points indicate farms. Point color and ellipses indicate ecoregion of farm: SE Coastal Plains (orange, dashed), Appalachian Mountains (blue, dotted), and Piedmont (green, solid). Species indicated in red were the sources of crop-collected fecal samples, as determined from DNA barcoding (see [20] for details).

The bird community NMDS had a two-axis solution (stress = 0.19, r^2^ = 0.96). The Appalachian Mountain and Coastal Plains regions clustered furthest away from each other, with the Piedmont region overlapping both. This corresponds to the latitudinal gradient of the area, although bird species were not noticeably clustered in any particular way between ecoregions (Figure 2), and bird community structure was not significantly associated with ecoregion (*p* > 0.05; Table S4). Furthermore, Mantel tests indicated that community structure was not associated with proportion developed, natural, agricultural, open water, or wetland land cover (*p* > 0.05; Table S5).

Using an indicator species analysis, we identified species that were significantly associated with certain ecoregions. The Western Cattle Egret (*Ardea ibis*) was associated with the Coastal Plains ecoregion (*p* = 0.05), and the Eastern Phoebe (*Sayornis phoebe*; *p* = 0.02), Eastern Towhee (*Pipilo erythrophthalmus*; *p* = 0.02), Indigo Bunting (*Passerina cyanea*; *p* = 0.001), Mourning Dove (*p* = 0.04), Song Sparrow (*Melospiza melodia*; *p* = 0.001), and Red-shouldered Hawk (*Buteo lineatus*; *p* = 0.03) were associated with the Appalachian Mountain ecoregion.

### 3.2. Factors Associated with Salmonella Prevalence

*Salmonella* was detected by culture or PCR in 8.6% (60/700) of total foliage-collected bird samples. We detected *Salmonella* in 1.6% (11/700) of samples by culture while 7% (49/700) additional samples were identified by PCR. We had five well-supported models linking livestock and landscape variables to *Salmonella* prevalence (Table S6). The interaction between on-farm cattle and wetlands was in three of the five models, while the interaction between on-farm chickens and natural land cover was in two of the five. Other livestock, i.e., the presence of livestock on-farm that were not chickens or cattle, was in two of the five models.

**Figure 3:**
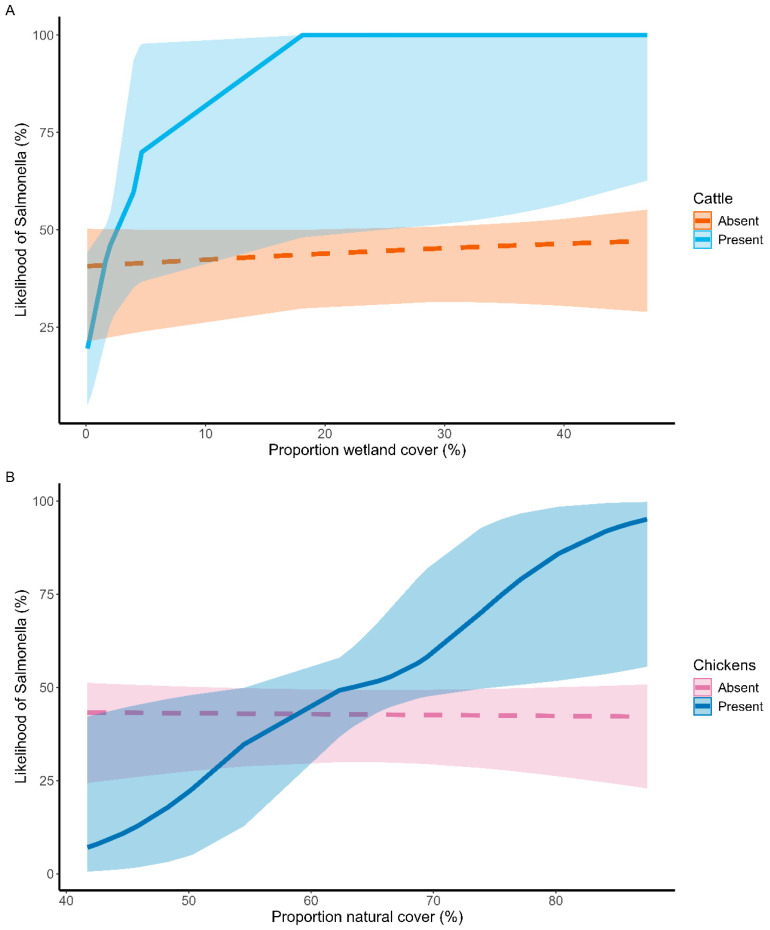
Salmonella prevalence was best predicted by the interaction between (**A**) on-farm cattle presence and a high proportion of wetland cover and (**B**) on-farm chicken presence and a high proportion of natural cover. Slopes come from top models as determined by AICc ± 95% CI. Color and line-type indicates (**A**) presence (blue, solid) or absence (red, dashed) of on-farm cattle and (**B**) presence (dark blue, solid) or absence (pink, dashed) of on-farm chickens.

As the proportion of wetland cover increased around farms, the presence of cattle on-farm was positively correlated with *Salmonella* prevalence in bird feces. The predicted likelihood of detecting *Salmonella* in bird feces increased from 25% to 99% when cattle were present on-farm at a proportion of wetland cover of 18% (Figure 3). Farms embedded in landscapes with a lower proportion of natural cover were more likely to have *Salmonella* detected in bird feces if they lacked chickens on-farm, compared to farms that had chickens. However, at higher proportions of natural landscape cover, this pattern was reversed, and *Salmonella* was more likely to be detected in feces from farms that had chickens. The predicted likelihood of detecting *Salmonella* in bird feces increased from 23% to 43% when chickens were present on-farm at a proportion of natural land cover of 66% (Figure 3).

## 4. Discussion

The Appalachian Mountain, Piedmont, and Coastal Plains ecoregions differ broadly in climate, topography, soil types, human land use, and plant communities, and correspond to three distinct Bird Conservation Regions [21]. Thus, it is perhaps surprising that we failed to find significant differences in the bird communities detected in produce fields in these different ecoregions (Figure 2). One possible explanation is that farming landscapes, regardless of the ecoregion where they are embedded, primarily attract and harbor the same synanthropic bird species tolerant of disturbance and/or that benefit from the open habitats that farming provides [36]. Overall, a fifth of the farms we surveyed were over 40 ha. (Table 1). Large, monoculture farms tend to have the same widely distributed, generalist species, even in different regions and landscapes [37]. Consistent with this possible explanation, many of the most common bird species observed in our Southeastern produce fields are the same as those observed in better studied western US produce farming systems: Song Sparrows (*Melospiza melodia*), Barn Swallows, and House Finches were all among the most commonly recorded species in our and similar west coast studies [10,11,38]. Likewise, in a survey of north-central Florida farms, Jones et al. (2005) [39] recorded the Northern Cardinal (*Cardinalis cardinalis*) and Northern Mockingbird (*Mimus polyglottos*)—the two species we also observed at the most farms—twice as frequently as the next most common species, and found that these two birds were associated with monoculture farms.

We found that *Salmonella* prevalence in foliage-collected bird feces was best predicted by two different interactions: it was higher when (1) cattle were observed on the farm and farms were surrounded by wetlands, and (2) chickens were observed on the farm and farms were surrounded by natural habitat (Figure 3). Both wetlands and livestock are known to be reservoirs for *Salmonella* [40,41,42,43]. Smith et al. (2023) [20] used whole-genome sequencing to characterize 19 *Salmonella* isolates from the same bird fecal samples analyzed in our study, and searched for genetic similarities to *Salmonella* isolates previously linked to various environmental (e.g., rivers and ponds) and agricultural (e.g., cattle or poultry production facilities) bacterial reservoirs. Interestingly, ref. [20] found that four out of the 19 avian-feces-derived *Salmonella* isolates were most closely related to isolates previously collected from surface water, consistent with the positive correlation we found between wetland prevalence in the landscape and *Salmonella* prevalence in surface-collected bird feces (Figure 3). Several foodborne *Salmonella* outbreaks that impacted human health have been linked to contamination of nearby water sources [44,45], including a significant outbreak in cucumbers in 2024 [46]. We identified several *Salmonella*-positive samples from our plant-surface collections as having been deposited by the wetland-associated Western Cattle Egret and Fish Crow (*Corvus ossifragus*) [20]. In a similar whole genome sequencing study, Fu et al. (2022) [47] found that *Salmonella* lineages collected from wading birds were most genetically similar to isolates from water sources. Additionally, Gorski et al. (2011) [48] linked *Salmonella* isolates found in surface water to those recovered from several species of wild birds, including crows (*Corvus* spp.).

Despite our finding that on-farm cattle and chickens were predictors of *Salmonella* prevalence (Figure 3), Smith et al. (2023) [20] found that none of the 19 sequenced *Salmonella* isolates were closely related to *Salmonella* isolated from cattle. Three isolates were somewhat similar to isolates from chickens, but the chicken isolates were from geographically distant states. This puzzling result, and previous work elsewhere that has linked *Salmonella* from livestock to that found in wild birds (e.g., [49]), could have several non-mutually exclusive explanations. Livestock and their associated habitat may draw in competent *Salmonella* reservoirs like Western Cattle Egrets, heightening food safety risks in nearby areas [50], even if the livestock themselves are not the source of the pathogens. In that case, there would be an apparent correlation between livestock and heightened food safety risks, but the livestock would only be causing an aggregation of hosts. For example, Pao et al. (2014) [51] recovered the same *Campylobacter* strain from different species of wild birds trapped at different ruminant pastures, but found that there was no transmission of bacteria between birds and ruminants. Phalen et al. (2010) [52] found that Western Cattle Egrets and horses had the same *Salmonella* serovars, but that these bacteria came from a common source and were likely not transmitted between species. Another possible explanation is that the livestock near our produce fields were grazing in open pasture or coops at relatively low densities. Conversely, most databank sequences (which were used in the Smith et al. (2023) [20] study) originate from regulatory samples collected from slaughterhouses or from animals grown in high-density feedlots, dairies, and broiler houses [53,54,55,56,57,58]. That is, the prior analysis on isolates from farms in this study may have missed a link between low-density, pastured livestock and *Salmonella* found in bird feces because the databank isolates they made comparisons to are biased towards sequences from high-density processing facilities. Clearly, more work is needed to detail where specifically wild birds are acquiring the potential foodborne pathogens that they later might spread to fresh produce fields, beyond the broad habitat associations reported here and elsewhere (e.g., [10,11,22]).

The ecology of foodborne pathogen dissemination by wild birds has been most intensively studied in agricultural production areas in the western US [9,10,13,49,59]. In several respects, we found patterns in our southeastern produce fields that broadly mirror previous results. In the west, as in our study region, livestock production, and particularly cattle production, was correlated with increased prevalence of potential foodborne pathogens in bird feces deposited on crops [10,11]. This suggests that produce growers in both regions that also raise livestock or are surrounded by livestock production should consider utilizing mitigation efforts to decrease bird-associated food safety risks [60].

We also found several key differences from what has been reported from previous studies in the western US. Most dramatically, whereas in the western fields *Campylobacter* spp. were by far the most common detected foodborne bacteria in bird feces, detected in ca. 3–13% of bird fecal samples [4,10,11], we never detected any *Campylobacter* from the feces collected in the Southeast. In stark contrast, we detected *Salmonella* in 1.6% of the bird fecal samples by culture from southeastern produce fields, whereas these bacteria were only found in ≤0.5% of western bird feces [4,10,11,48,49,61]. It is unclear whether this difference between regions reflects true differences in prevalence of the pathogen taxa (*Campylobacter* versus *Salmonella*), differences in the importance or abundance of surrounding wetlands, or some other factor such as regional differences in presence of competent avian hosts. The geographically closest equivalent studies we could find come from southeast Texas, where Grigar et al. (2017) [62] reported *Salmonella* rates of 0.5% in waterfowl taken from the Gulf Coast, while Brobey et al. (2017) [63] found *Salmonella* rates of 17% in wild birds trapped from suburban and urban sites. In Florida, Hernandez et al. (2016) [18] reported a *Salmonella* prevalence of 13% from White Ibis (*Eudocimus albus*). It is also important to note that some of our comparisons of bacterial prevalence to studies in the western US are limited due to differences in how fecal samples were collected and stored, which could have differently affected *Campylobacter* and *Salmonella* survival and detection. We also used a *Campylobacter* PCR method that targeted the 16S gene, which differs from some previous studies. Overall, our findings suggest the need for more comparative studies in the ecology of bird-associated food safety risks between regions, using identical methods, to determine what specific regional and environmental differences best explain pathogen prevalence in agroecosystems.

Given the absence of *Campylobacter* detection in bird feces here, we suggest that birds may pose a limited food safety risk in terms of bacterial transmission to produce. This study suggests that one of the primary risks is transmission by birds between livestock and crops on the same farm. Growers could help mitigate this transmission pathway by using physical barriers like netting or grates to keep birds out of livestock barns or feed storage. Smith et al. (2023) [20] found that there was no transmission of pathogenic bacteria from crop-deposited feces to produce on the same plant or plants downwind from feces, further suggesting that food safety risks from birds are generally low, at least in the produce fields sampled. Future studies may want to consider bacterial transmission to produce elsewhere in the harvesting and packing process—for example, birds nesting above food packaging areas—as potentially bigger food safety risks.

## 5. Conclusions

The results presented here suggest several next steps for better understanding how natural habitats on or near farms interact with regional bird communities to determine birds’ threat to food safety. First, it appears that there may be some similarities, but many key differences, between US regions in landscape drivers of bird-associated food safety risks and in the pathogen taxa most likely to be transmitted. This primarily suggests the need for more widespread studies into the ecology of birds and food safety in other important produce growing regions in North America and elsewhere. Additionally, complementary genetic inference of pathogen sources in Smith et al. (2023) [20] matches the landscape correlates we found in terms of wetlands appearing as both a risk factor associated with *Salmonella* detection in bird feces and as the likely environmental source of these bacteria. Yet, while livestock appeared as clear correlates of pathogen detection in bird feces here and in the west [10,11], genetic tracking did not detect *Salmonella* isolates typically found in cattle nor chickens [20]. This suggests the need for more work directly linking habitats to potential foodborne pathogens that birds may be exposed to, in many different produce growing regions. Further, we suggest a clear need to identify specific links between a given level of pathogen prevalence in bird feces and the later risk to human consumers. It is likely that temperature, humidity, irrigation practices, etc., all impact pathogen persistence after fecal deposition, although these effects are relatively understudied (but see [59,61,64]). Finally, we know that birds can contribute to natural control of insect and rodent pests of agriculture [65], and also weed control [66], but relatively few studies simultaneously look at both ecosystem services and disservices provided by birds in the same cropping fields (but see [4,9,67]). Ultimately, it is the balance of these beneficial and harmful impacts of birds that will determine whether productive farming and bird conservation can be compatible goals.

## Figures and Tables

**Table 1:** The number of farms (# Farms) with each characteristic by state (TN = Tennessee, GA = Georgia, SC = South Carolina, FL = Florida). “Size” refers to farm size. Livestock were counted as present if they were within 250 m of the farm during at least one survey period. “Other” includes all other livestock species, i.e., horse, goat, llama, pig, donkey, and duck.

	TN	GA	SC	FL	Total
# Farms	4	26	10	3	43
0–4 ha	0	6	2	0	8
4.1–20 ha	1	12	4	1	18
20.1–40 ha	0	7	2	1	10
40+ ha	3	1	2	1	7
Cattle	3	7	0	1	11
Chicken	2	10	3	0	15
Other	1	3	3	0	7
Monoculture	3	19	2	2	26
Mixed crops	1	7	8	1	17

## Data Availability

The original data and R code presented in the study are openly available on GitHub at https://doi.org/10.5281/zenodo.16762320.

## Supplementary Materials

The following supporting information can be downloaded at: https://www.mdpi.com/article/10.3390/ani15192813/s1, Table S1: Landscape and livestock factors; Figure S1: Pearson correlation values; Table S2: List of generalized linear effects models; Table S3: Total number of individuals of each bird species; Table S4: R statistics and *p* values for bird community ANOSIM tests; Table S5: R statistics and *p* values for bird community Mantel tests; Table S6: Model estimates.
