# Supplementary material for: Wild Birds Pose Unique Food Safety Threats in the US Southeast
Source: Animals (Basel). 2025 Sep 26;15(19):2813. doi: 10.3390/ani15192813 (PMC12523605; doi:10.3390/ani15192813)
Supplement: Supplementary file 1 [file animals-15-02813-s001.zip › animals-3835857-supplementary.pdf]

Supplementary Materials

Table S1. Landscape and livestock factors examined in relation to *Salmonella* prevalence. Values are mean and (min-max values). We selected which natural cover configurational and compositional heterogeneity variables to include in models based on their Pearson's correlation coefficients (Supplementary Material Figure S1). Landscape metrics use a 4.5 km radius.

Category	Metric Type	Class	Used in analysis	Definition
% Open Water	landscape composition	percentage mean: 2.98 (0.1-19.52)	yes	Proportion of open water
% Developed	landscape composition	percentage mean: 11.21 (2.22-42.04)	yes	Proportion of developed habitat (NLCD codes 21-24)
% Barren	landscape composition	percentage mean: 0.19 (0-1.02)	no	Proportion barren habitat (NLCD code 31)
% Agricultural	landscape composition	percentage mean: 18.12 (2.65-47.06)	yes	Proportion of agricultural habitat (NLCD codes 81-82)
% Natural	landscape composition	percentage mean: 67.40 (41.71-92.49)	yes	Proportion of natural habitat (NLCD codes 41-743, 51-52, 71-74, 90, 95)
% Wetland	landscape composition	percentage mean: 24.28 (0.13-54.23)	yes	Proportion of wetland (NLCD codes 90, 95)
Nat_ED	configurational	numeric mean: 45.08 (18.89-74.00)	no	Edge Density of natural habitat
Nat_AREA_AM	configurational	numeric mean: 3352.34 (252.64-5883.01)	no	Area weighted mean patch area
Nat_ENN_AM	configurational	numeric mean: 59.93 (59.33-61.63)	no	Euclidean Nearest Neighbor Distance
Nat_IJI	configurational	numeric mean: 62.05 (50.92-84.50)	yes	Interspersion and Juxtaposition Index
CONTAG	configurational	numeric mean: 62.31	no	Contagion Index across all land cover types

		(41.74-85.39)		
SHDI	compositional	numeric<br>mean: 0.82<br>(0.32-1.23)	no	Shannon's Habitat Diversity Index
SHEI	compositional	numeric<br>mean: 0.51<br>(0.20-0.77)	no	Shannon's Habitat Evenness Index
Chicken	on-farm livestock	presence/absence	yes	Chickens within 250 m
Cow	on-farm livestock	presence/absence	yes	Cows within 250 m
Other	on-farm livestock	presence/absence	yes	Other livestock within 250 m
Livestock	on-farm livestock	presence/absence	yes	Any livestock within 250 m

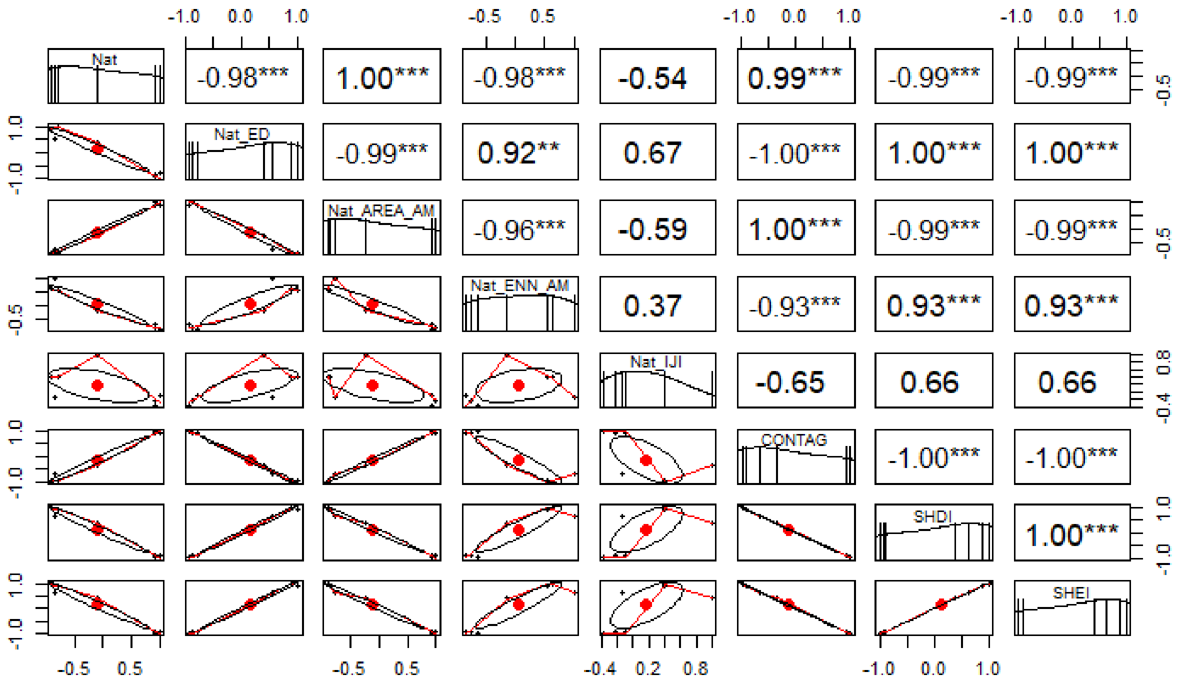

Figure S1. Pearson correlation values for proportion natural (NLCD) and calculated natural configurational and compositional heterogeneity in a 4.5 km radius around farms. Abbreviations used are defined in Table S3. Bolded values indicate correlations significant at p < 0.05; asterisks indicate correlations significant at p < 0.01.

Table S2. List of generalized linear mixed effects models used to test associations between landscape and on-farm livestock variables as predictors of *Salmonella* prevalence in foliage-collected bird feces. Refer to Table S1 for explanations of fixed effects. Year was included in all models as a fixed effect. (1|Farm/Visit) represents the random effect, wherein visit is nested within farm.

#	Model Structure
Null	Year + (1|Farm/Visit)
1	% Open Water + Year + (1|Farm/Visit)
2	% Developed + Year + (1|Farm/Visit)
3	% Agricultural + Year + (1|Farm/Visit)
4	% Natural + Year + (1|Farm/Visit)
5	% Wetland + Year + (1|Farm/Visit)
6	Nat_IJI + Year + (1|Farm/Visit)
7	Chicken + Year + (1|Farm/Visit)
8	Cow + Year + (1|Farm/Visit)
9	Other + Year + (1|Farm/Visit)
10	Livestock + Year + (1|Farm/Visit)
11	% Open Water + Chicken + Year + (1|Farm/Visit)
12	% Developed + Chicken + Year + (1|Farm/Visit)
13	% Agricultural + Chicken + Year + (1|Farm/Visit)
14	% Natural + Chicken + Year + (1|Farm/Visit)
15	% Wetland + Chicken + Year + (1|Farm/Visit)
16	Nat_IJI + Chicken + Year + (1|Farm/Visit)
17	% Open Water * Chicken + Year + (1|Farm/Visit)
18	% Developed * Chicken + Year + (1|Farm/Visit)
19	% Agricultural * Chicken + Year + (1|Farm/Visit)
20	% Natural * Chicken + Year + (1|Farm/Visit)
21	% Wetland * Chicken + Year + (1|Farm/Visit)
22	Nat_IJI * Chicken + Year + (1|Farm/Visit)
23	% Open Water + Cow + Year + (1|Farm/Visit)
24	% Developed + Cow + Year + (1|Farm/Visit)
25	% Agricultural + Cow + Year + (1|Farm/Visit)
26	% Natural + Cow + Year + (1|Farm/Visit)
27	% Wetland + Cow + Year + (1|Farm/Visit)
28	Nat_IJI + Cow + Year + (1|Farm/Visit)
29	% Open Water * Cow + Year + (1|Farm/Visit)
30	% Developed * Cow + Year + (1|Farm/Visit)
31	% Agricultural * Cow + Year + (1|Farm/Visit)
32	% Natural * Cow + Year + (1|Farm/Visit)
33	% Wetland * Cow + Year + (1|Farm/Visit)
34	Nat_IJI * Cow + Year + (1|Farm/Visit)

35	% Open Water + Other + Year + (1|Farm/Visit)
36	% Developed + Other + Year + (1|Farm/Visit)
37	% Agricultural + Other + Year + (1|Farm/Visit)
38	% Natural + Other + Year + (1|Farm/Visit)
39	% Wetland + Other + Year + (1|Farm/Visit)
40	Nat_IJI + Other + Year + (1|Farm/Visit)
41	% Open Water * Other + Year + (1|Farm/Visit)
42	% Developed * Other + Year + (1|Farm/Visit)
43	% Agricultural * Other + Year + (1|Farm/Visit)
44	% Natural * Other + Year + (1|Farm/Visit)
45	% Wetland * Other + Year + (1|Farm/Visit)
46	Nat_IJI * Other + Year + (1|Farm/Visit)
47	% Open Water + Livestock + Year + (1|Farm/Visit)
48	% Developed + Livestock + Year + (1|Farm/Visit)
49	% Agricultural + Livestock + Year + (1|Farm/Visit)
50	% Natural + Livestock + Year + (1|Farm/Visit)
51	% Wetland + Livestock + Year + (1|Farm/Visit)
52	Nat_IJI + Livestock + Year + (1|Farm/Visit)
53	% Open Water * Livestock + Year + (1|Farm/Visit)
54	% Developed * Livestock + Year + (1|Farm/Visit)
55	% Agricultural * Livestock + Year + (1|Farm/Visit)
56	% Natural * Livestock + Year + (1|Farm/Visit)
57	% Wetland * Livestock + Year + (1|Farm/Visit)
58	Nat_IJI * Livestock + Year + (1|Farm/Visit)
59	Chicken + Cow + Year + (1|Farm/Visit)
60	Chicken + Other + Year + (1|Farm/Visit)
61	Cow + Other + Year + (1|Farm/Visit)
62	Chicken * Cow + Year + (1|Farm/Visit)
63	Chicken * Other + Year + (1|Farm/Visit)
64	Cow * Other + Year + (1|Farm/Visit)
65	% Open Water * Chicken + Cow + Year + (1|Farm/Visit)
66	% Developed * Chicken + Cow + Year + (1|Farm/Visit)
67	% Agricultural * Chicken + Cow + Year + (1|Farm/Visit)
68	% Natural * Chicken + Cow + Year + (1|Farm/Visit)
69	% Wetland * Chicken + Cow + Year + (1|Farm/Visit)
70	Nat_IJI * Chicken + Year + Cow + Year + (1|Farm/Visit)
71	% Open Water * Cow + Chicken + Year + (1|Farm/Visit)
72	% Developed * Cow + Chicken + Year + (1|Farm/Visit)
73	% Agricultural * Cow + Chicken + Year + (1|Farm/Visit)
74	% Natural * Cow + Chicken + Year + (1|Farm/Visit)
75	% Wetland * Cow + Chicken + Year + (1|Farm/Visit)
76	Nat_IJI * Cow + Chicken + Year + (1|Farm/Visit)

77	% Open Water + Cow * Chicken + Year + (1|Farm/Visit)
78	% Developed + Cow * Chicken + Year + (1|Farm/Visit)
79	% Agricultural + Cow * Chicken + Year + (1|Farm/Visit)
80	% Natural + Cow * Chicken + Year + (1|Farm/Visit)
81	% Wetland + Cow * Chicken + Year + (1|Farm/Visit)
82	Nat_IJI + Cow * Chicken + Year + (1|Farm/Visit)
83	% Open Water * Chicken + Other + Year + (1|Farm/Visit)
84	% Developed * Chicken + Other + Year + (1|Farm/Visit)
85	% Agricultural * Chicken + Other + Year + (1|Farm/Visit)
86	% Natural * Chicken + Other + Year + (1|Farm/Visit)
87	% Wetland * Chicken + Other + Year + (1|Farm/Visit)
88	Nat_IJI * Chicken + Other + Year + (1|Farm/Visit)
89	% Open Water * Other + Chicken + Year + (1|Farm/Visit)
90	% Developed * Other + Chicken + Year + (1|Farm/Visit)
91	% Agricultural * Other + Chicken + Year + (1|Farm/Visit)
92	% Natural * Other + Chicken + Year + (1|Farm/Visit)
93	% Wetland * Other + Chicken + Year + (1|Farm/Visit)
94	Nat_IJI * Other + Chicken + Year + (1|Farm/Visit)
95	% Open Water + Other * Chicken + Year + (1|Farm/Visit)
96	% Developed + Other * Chicken + Year + (1|Farm/Visit)
97	% Agricultural + Other * Chicken + Year + (1|Farm/Visit)
98	% Natural + Other * Chicken + Year + (1|Farm/Visit)
99	% Wetland + Other * Chicken + Year + (1|Farm/Visit)
100	Nat_IJI + Other * Chicken + Year + (1|Farm/Visit)
101	% Open Water * Cow + Other + Year + (1|Farm/Visit)
102	% Developed * Cow + Other + Year + (1|Farm/Visit)
103	% Agricultural * Cow + Other + Year + (1|Farm/Visit)
104	% Natural * Cow + Other + Year + (1|Farm/Visit)
105	% Wetland * Cow + Other + Year + (1|Farm/Visit)
106	Nat_IJI * Cow + Other + Year + (1|Farm/Visit)
107	% Open Water * Other + Cow + Year + (1|Farm/Visit)
108	% Developed * Other + Cow + Year + (1|Farm/Visit)
109	% Agricultural * Other + Cow + Year + (1|Farm/Visit))
110	% Natural * Other + Cow + Year + (1|Farm/Visit)
111	% Wetland * Other + Cow + Year + (1|Farm/Visit))
112	Nat_IJI * Other + Cow + Year + (1|Farm/Visit)
113	% Open Water + Other * Cow + Year + (1|Farm/Visit)
114	% Developed + Other * Cow + Year + (1|Farm/Visit)
115	% Agricultural + Other * Cow + Year + (1|Farm/Visit)
116	% Natural + Other * Cow + Year + (1|Farm/Visit)
117	% Wetland + Other * Cow + Year + (1|Farm/Visit)
118	Nat_IJI + Other * Cow + Year + (1|Farm/Visit)

Table S3. Total percentages of individuals of each bird species recorded during point count surveys. Total % gives the total of all ecoregions; % AM the percentage out of bird species recorded in the Appalachian Mountains region; % P the percentage out of bird species recorded in the Piedmont region; and % SCP the percentage of birds recorded in the Southeastern Coastal Plains region.

Scientific Name	Common Name	Total %	% AM	% P	% SCP
Buteo jamaicensis	Red-tailed Hawk	0.12	0	0.23	0
Buteo lineatus	Red-shouldered Hawk	0.70	0.71	0.93	0
Chaetura pelagica	Chimney Swift	0.81	0	0.23	4.11
*Archilochus colubris**	Ruby-throated Hummingbird	0.12	0	0	0.68
Coragyps atratus	Black Vulture	1.05	0	2.09	0
*Charadrius vociferous**	Killdeer	0.93	1.06	0.47	2.05
*Columba livia**†	Rock Pigeon	12.46	37.10	0.47	0
*Columbina passerina**	Common Ground Dove	0.35	0	0.93	0
*Streptopelia decaocto**†	Eurasian Collared-Dove	0.70	0.35	0.47	2.05
*Zenaida macroura**	Mourning Dove	12.57	18.37	8.60	13.01
*Cardinalis cardinalis**	Northern Cardinal	5.94	0	8.14	10.96
*Passerina caerulea**	Blue Grosbeak	0.93	0.71	1.40	0
Passerina ciris	Painted Bunting	0.23	0	0	1.37
*Passerina cyanea**	Indigo Bunting	0.47	1.41	0	0
*Corvus brachyrhynchos**	American Crow	3.49	3.53	3.95	2.05
*Cyanocitta cristata**	Blue Jay	1.63	1.06	2.56	0
*Haemorhous mexicanus**†	House Finch	7.10	0.71	10.47	9.59
*Spinus tristis**	American Goldfinch	0.81	0.35	1.40	0
*Hirundo rustica**	Barn Swallow	7.80	7.42	7.44	9.59
Stelgidopteryx serripennis	Northern Rough-winged Swallow	0.47	0	0.93	0
Tachycineta bicolor	Tree Swallow	0.12	0.35	0	0
*Agelaius phoeniceus**	Red-winged Blackbird	1.63	1.06	0.47	6.16
Quiscalus quiscula	Common Grackle	0.35	0	0.47	0.68
*Sturnella magna**	Eastern Meadowlark	0.47	1.41	0	0
*Mimus polyglottos**	Northern Mockingbird	5.70	0.35	7.91	9.59
*Toxostoma rufum**	Brown Thrasher	0.47	0.35	0.47	0.68
*Baeolophus bicolor**	Tufted Titmouse	0.93	0	1.86	0
Poecile carolinensis	Carolina Chickadee	1.28	0.35	2.32	0
Setophaga americana	Northern Parula	0.12	0.35	0	0
Setophaga pinus	Pine Warbler	0.12	0	0.23	0
*Melospiza melodia**	Song Sparrow	3.61	10.25	0.47	0
Pipilo erythrophthalmus	Eastern Towhee	1.40	1.06	1.86	0.68

*Spizella passerine**	Chipping Sparrow	4.77	3.18	6.98	1.37
*Spizella pusilla**	Field Sparrow	1.05	2.12	0.70	0
Passer domesticus†	House Sparrow	0.12	0	0.23	0
*Sturnus vulgaris**†	European Starling	1.86	0	3.72	0
*Thryothorus ludovicianus**	Carolina Wren	4.42	0.71	7.44	2.74
*Sialia sialis**	Eastern Bluebird	3.84	1.41	4.65	6.16
Turdus migratorius	American Robin	0.58	1.77	0	0
*Sayornis phoebe**	Eastern Phoebe	3.26	1.77	5.12	0.68
*Tyrannus tyrannus**	Eastern Kingbird	0.47	0	0	2.74
Vireo flavifrons	Yellow-throated Vireo	0.23	0.35	0.23	0
*Vireo griseus**	White-eyed Vireo	1.28	0.35	2.09	0.68
*Ardea ibis**	Western Cattle Egret	2.10	0	0	12.33
*Dryobates pubescens**	Downy Woodpecker	0.58	0	1.16	0
Dryocopus pileatus	Pileated Woodpecker	0.12	0	0.23	0
Melanerpes carolinus	Red-bellied Woodpecker	0.35	0	0.70	0

*Denotes which species were observed interacting with crops (e.g., perched on trellises, entering grow tunnels); for total number of contacts per species see Smith et al. (2023)[20].
†Denotes which species are non-native.

Table S4. R statistics and *p* values for bird community ANOSIM tests. The "strata" column defines the strata used in the function *anosim* from the *vegan* R package. "Livestock presence" refers to whether any livestock were present on a farm. Each row refers to a separate test, with permutations within each stratum, which were tested separately due to the low number of farms at some values.

Model	Strata	R	p
Ecoregion	field size	0.05	0.24
Ecoregion	livestock presence	0.05	0.25

Table S5. R statistics and *p* values for bird community Mantel tests that examined relationships between land cover variables and Bray–Curtis dissimilarity matrices. The "strata" column defines the strata used in the function *mantel* from the *vegan* R package. "Livestock presence" refers to whether any livestock were present on a farm. Each row refers to a separate test, with permutations within each stratum, which were tested separately due to the low number of farms at some values.

Model	Strata	R	p
Proportion Developed Land	field size	-0.07	0.81
Proportion Developed Land	livestock presence	-0.07	0.90
Proportion Natural Land	field size	-0.04	0.75
Proportion Natural Land	livestock presence	-0.04	0.71
Proportion Agricultural Land	field size	0.06	0.14
Proportion Agricultural Land	livestock presence	0.06	0.19
Proportion Wetlands	field size	0.02	0.31
Proportion Wetlands	livestock presence	0.02	0.09

Table S6. Model estimates with values ± 95% confidence intervals (CIs) in columns. Only those with > 5% of AIC_c model weight are shown. "Wet" and "Nat" refer to proportion of wetlands and natural habitat, respectively, in a 4.5 km radius around a farm. "Cattle, "Chick", and "Other" refer to whether cattle, chickens, or other livestock, respectively, were present on a farm. Bolded values indicate values where 95% CIs do not overlap 0.

Model	Wet	Cattle	Wet:Cow	Nat	Chick	Nat:Chick	Other	Year	ΔAIC_c	AIC_c Weight
Wet * Cattle + Year	0.16 ± 4.66	4.35 ± 4.43	**11.38 ± 10.83**					**-1.07 ± 1.02**	0.00	0.22
Wet * Cattle + Chick + Year	0.22 ± 0.50	4.16 ± 4.47	**11.01 ± 10.89**		0.65 ± 1.09			-1.10 ± 1.10	0.71	0.15
Nat * Chick + Other + Year				-0.03 ± 0.56	0.35 ± 1.07	**2.52 ± 1.77**	-0.90 ± 1.23	**-1.03 ± 0.93**	1.89	0.08
Nat * Chick + Year				-0.12 ± 0.58	0.18 ± 1.11	**2.31 ± 1.68**		**-1.28 ± 0.96**	1.90	0.08
Wet * Cattle + Other + Year	0.14 ± 0.48	4.40 ± 4.45	**11.44 ± 10.87**				-0.17 ± 1.24	**-1.05 ± 1.01**	2.05	0.08
